# Surgical Site Infection Following Dermatologic and Plastic Surgery in an Office Setting

**DOI:** 10.7759/cureus.88349

**Published:** 2025-07-20

**Authors:** Daichi Morioka, Satoru Tamura, Takaharu Otsuka, Sayo Tatsuta, Mari Nakahara

**Affiliations:** 1 Department of Plastic Surgery, Kitasenju Clinic of Plastic Surgery and Dermatology, Tokyo, JPN; 2 Department of Plastic Surgery, National Defense Medical College Hospital, Saitama, JPN; 3 Department of Plastic Surgery, Showa Medical University Fujigaoka Hospital, Kanagawa, JPN

**Keywords:** dermatologic surgery, office setting, outpatient, plastic surgery, skin tumor, surgical site infection

## Abstract

Background: Surgical site infections (SSI) in skin surgery are rare but can cause significant patient discomfort and increase treatment costs.

Objective: To evaluate the incidence and risk factors of SSIs in outpatient dermatologic and plastic surgeries and to highlight effective prevention strategies.

Methods: We conducted a retrospective chart review of outpatient performed procedures between 2012 and 2024. The inclusion criteria were Class I (clean) and Class II (clean-contaminated) surgeries, which required wound approximation.

Results: Among the 8,742 procedures performed during the study period, seven infections (0.08%) were identified, with significantly higher rates in complex procedures (1.42%) than in simple closures (0.06%). The hair-bearing regions showed a trend toward a high infection risk.

Conclusions: Although a single-center, retrospective study, it included a large sample size with a broad age distribution and representation of various anatomical areas. The incidence of SSIs in this study was extremely low compared with previous studies. We emphasize the importance of proper surgical techniques, cautious use of subcuticular sutures for hair-bearing regions, and comprehensive patient education on postoperative care.

## Introduction

Wound infections following skin surgery and minor plastic surgery conducted in outpatient settings are generally rare and typically non-life-threatening [1,2]. However, office-based dermatologic procedures have become increasingly prevalent over the years [3], and the absolute number of patients experiencing postoperative wound infections is presumed to rise proportionally. Although uncommon, when these infections occur, they can significantly impair wound healing, cause considerable patient discomfort, and increase morbidity, consequently leading to heightened treatment costs [4]. Additionally, poor cosmetic outcomes and scar contractures can adversely affect patients’ psychosocial well-being [5].

According to a recent systematic review by Schlager et al., the incidence of surgical site infections (SSIs) in dermatological procedures ranges from 0.96% to 8.7% [4]. However, most studies conducted to date have been small-scale (involving fewer than 2,000 patients or procedures), resulting in underpowered SSI rate estimates and varied reports of associated risk factors [1,2,4,6]. Since the principal author (D.M.) established a private outpatient dermatologic and plastic surgery clinic in downtown Tokyo in 2012, more than 15,000 procedures have been performed under local anesthesia. These procedures include abscess drainage, superficial trauma management, skin and subcutaneous tumor excision, ingrown toenail treatment, tattoo removal, and minor aesthetic surgeries, such as blepharoplasty and nipple reduction. Over 12 years of private practice, wound infections following surgical procedures for Class I or II conditions have rarely occurred.

Two primary categories of risk factors influencing SSIs following dermatologic and plastic surgeries have been frequently reported in the literature: patient-related and surgery-related [5,7,8]. Patient-related factors include male sex, malignancy, and comorbidity [4,8,9]. High-risk anatomical sites have varied across the studies, potentially due to differences in sample sizes, etiologies, and clinical settings; however, frequently implicated areas include the scalp, ears, arms, genitalia, and lower extremities [1,2,8,10]. Surgery-related risk factors include surgical duration, defect size, and the complexity of surgical techniques [2,8]. Furthermore, variations in SSI incidence may also be attributed to differences in study definitions of SSI [5]. While most prior studies have utilized definitions established by the European Centre for Disease Prevention and Control or the Centers for Disease Control and Prevention (CDC) [11,12], others have applied non-standard definitions [9,13] or provided none at all [14].

In this study, we aimed to determine the incidence of SSIs according to CDC guidelines [15] and to identify potential risk factors for SSIs through a retrospective review of medical charts from a large cohort of outpatient dermatologic and plastic surgeries. Additionally, we aimed to elucidate the details of our operative techniques and discuss strategies for SSI prevention in office-based skin surgeries, acknowledging that previous studies often lacked detailed descriptions of their surgical methodologies.

This article was previously presented as a meeting abstract at the 42nd Japanese Society for Dermatologic Surgery Annual Meeting on February 22, 2025.

## Materials and methods

Ethical approval

Ethical approval was obtained from the Ethical Review Committee of the National Defense Medical College (Approval No. 5047).

Patients and conditions

Medical charts and operative records of patients who underwent dermatologic, plastic, or aesthetic surgery at our clinic between May 2012 and November 2024 were retrospectively reviewed. Patients and procedures were selected based on the following inclusion criteria: (i) Class I (clean) or Class II (clean, contaminated) surgeries; (ii) conditions or pathologies including skin and soft tissue tumors, incisional biopsies for skin lesions, congenital anomalies, scar revisions, tattoo removal, and minor aesthetic surgeries, all of which required wound approximation; (iii) surgical techniques involving primary closure, local flaps, and skin grafting.

In cases involving multiple surgeries on a single patient, such as serial excision of a large nevus or repeated excision of multiple lipomatoses, the most significant procedure was selected for analysis. Therefore, the number of surgical procedures performed was equivalent to the total number of patients. Emergency trauma treatments, open procedures (such as dermabrasion for tattoos and shave biopsies), and Class III/IV surgeries for infected epidermoid cysts, pilonidal sinus, hidradenitis suppurativa, ingrown toenails, and pressure ulcers were excluded.

SSIs were defined according to the "superficial incisional SSI" criteria of the CDC guidelines, characterized by local symptoms indicative of infection (e.g., purulent discharge, increased pain, swelling, erythema, and/or heat) occurring within 30 days postoperatively, occasionally confirmed by positive culture results [15].

Procedures

All surgical procedures were performed under local anesthesia at the outpatient surgical unit of the Kitasenju Clinic of Plastic Surgery and Dermatology. Over 90% of the operations were conducted by the principal author (D.M.), and the remainder were performed by the coauthors. All procedures were performed by a single surgeon without a scrub assistant, supported by the circulating staff. Surgical loupes were used in most procedures except for extensive mass excisions.

**Figure 1:**
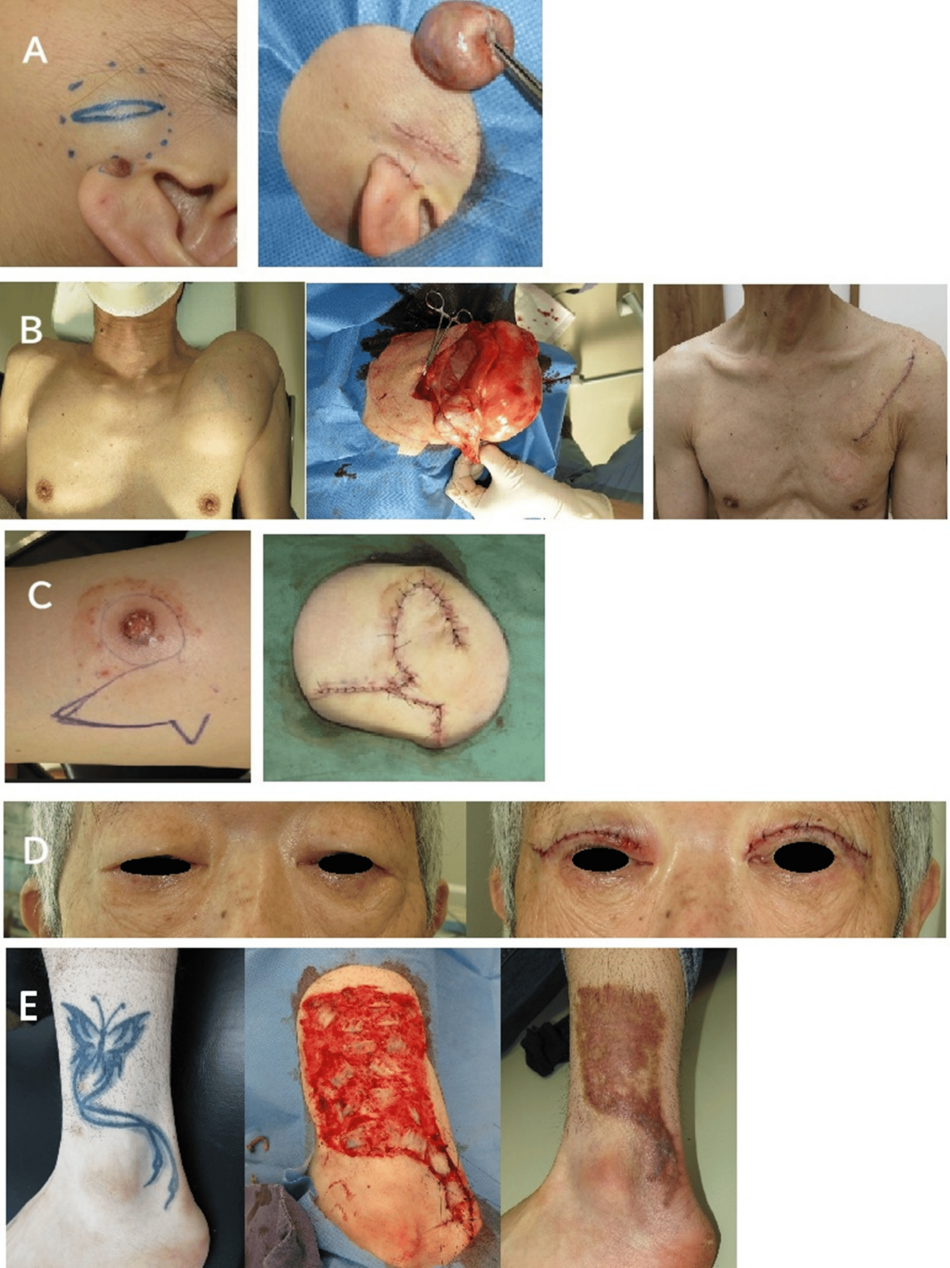
Representative cases of dermatologic and plastic surgery procedures A: Epidermoid cyst and intradermal nevus of the cheek (left) with the immediate postoperative appearance following simultaneous excisions (right). B: Subcutaneous giant lipoma of the chest (left), intraoperative view (center), and postoperative view one week after excision (right). C: Squamous cell carcinoma of the thigh (left) excised with a 2cm margin, with the resulting defect reconstructed using a fasciocutaneous transposition flap (right). D: Blepharoptosis (left) corrected via levator tucking and redundant skin excision (right). E: Tattoo of the lower leg (left) excised with defect closure achieved through postage stamp grafting and partial primary closure (center), and postoperative view one year after excision (right).

The surgical field was disinfected with 10% benzalkonium chloride for facial procedures and 0.5% chlorhexidine gluconate for other anatomical regions. Wound closure techniques included simple approximation, local flaps (e.g., rotation and transposition flaps), and full- or split-thickness skin grafting. Most wounds were closed using buried interrupted subcuticular sutures made of polyglactin or polydioxanone, followed by superficial suturing with nylon. Subcuticular sutures were not applied to skin grafts or wounds located on the scalp, eyelids, lips, hands, feet, or genitals. A Penrose drain was placed in the subcutaneous space when large soft-tissue tumors were excised or when postoperative hemorrhage was anticipated. In some instances, skin sutures were omitted and only subcuticular sutures were applied, particularly in the head and neck regions. The compression gauze dressings were typically applied overnight. Representative case photographs are described in Figure 1.

Perioperative oral antibiotics (amoxicillin or cefaclor) were administered to most patients for two to three days. Patients with Penrose drains were instructed to return for removal within two days postoperatively. The other patients were advised to perform wound care independently at home until suture removal. All patients received postoperative wound care instruction handouts detailing wound care, including maintaining wound closure for at least 12 hours, followed by daily dressing changes or open care with gentle cleansing until suture removal. Additionally, patients were educated regarding postoperative restrictions on physical activity and alcohol consumption to prevent complications and signs of infection, and the importance of promptly contacting the clinic if infection was suspected. Suture removal was scheduled between postoperative days 5-7 for the face and neck and days 10-20 for other body regions.

Data analysis

For analysis of surgical procedures, complex surgeries included split- or full-thickness skin grafting and skin flaps such as rotation, V-Y advancement, and transposition flaps. Superficial (in situ) skin cancers, such as Bowen’s disease, were classified as malignant skin tumors. Other plastic surgery cases included rhinoplasty, otoplasty (e.g., accessory auricles and cleft earlobe), and correction of the short labial frenum. Other conditions included minor skin ulcers primarily caused by third-degree burns, skin tears resulting from piercing, foreign body removal, or dermatitis biopsy. The statistical significance of the associations between categorical variables (e.g., sex and procedure type) was evaluated using Fisher’s exact test. Odds ratios (OR) and 95% confidence intervals (CI) were calculated. Data analysis was performed using EZR (Easy R) statistical software (version 1.68, Jichi Medical University, Saitama, Japan) [16]. A *P*-value <0.05 was considered statistically significant for all analyses.

## Results

A comprehensive review of medical charts and operative records identified 8,742 patients who met the inclusion criteria, with only one patient who underwent aesthetic otoplasty opting out of the study. Patient demographics and operative characteristics are summarized in Table 1. The cohort consisted of 4,787 female patients (54.8%) and 3,955 male patients (45.2%), with a mean age at the time of surgery of 42.2 years (SD, 20.5). The age distribution ranged from a one-year-old male infant presenting with a short upper lip frenum to a 99-year-old female patient with actinic keratosis of the cheek. Lesion sizes, measured by maximum diameter, ranged from 0.3 cm (nevus) to 23 cm (lipoma).

**Table 1:** Patient demographics and incidence of surgical site infections SSI, surgical site infection

Category	n (%)	SSI, n	p
Total	8742 (100)	7	-
Sex			
-Female	4787 (54.8)	2	0.26
-Male	3955 (45.2)	5	-
Procedure			
-Primary closure	8601 (98.4)	5	<0.01
-Complexed	141 (1.6)	2	-
Diagnosis/pathology			
-Benign tumors	7482 (85.6)	5	-
-Malignant tumors	115 (1.3)	-	-
-Blepharoplasty	285 (3.3)	-	-
-Scar revision	259 (3.0)	-	-
-Tattoo removal	139 (1.6)	1	-
-Osmidrosis	68 (0.8)	1	-
-Other plastic surgery	224 (2.6)	-	-
-Other skin conditions	170 (1.9)	-	-
Affected site			
-Scalp	298 (3.4)	-	-
-Forehead	339 (3.9)	-	-
-Eyelid	546 (6.2)	-	-
-Ear	553 (6.3)	-	-
-Cheek	880 (10.1)	-	-
-Nose	265 (3.0)	-	-
-Lip	138 (1.6)	-	-
-Chin	355 (4.1)	-	-
-Neck	836 (9.6)	2	-
-Upper extremity	1054 (12.1)	1	-
-Chest	304 (3.5)	1	-
-Back	1474 (16.9)	-	-
-Abdomen	254 (2.9)	1	-
-Buttock	484 (5.5)	-	-
-Genital region	382 (4.4)	-	-
-Lower extremity	580 (6.6)	2	-

Benign cutaneous or subcutaneous tumor excisions constituted the majority of cases, accounting for 85.6% (7,482 cases). Among these, cystic lesions, such as epidermoid and trichilemmal cysts, comprised more than half of the benign tumors, followed by nevi and lipomas. Malignant tumor excision represented only 1.3% (115 cases) of procedures, primarily including actinic keratosis, Bowen’s disease, and basal cell carcinoma. No cases of malignant melanoma were identified. Among the plastic surgical procedures, blepharoplasty was the most common (285 cases), followed by scar revision (259 cases) and tattoo removal (139 cases). Other minor procedures included rhinoplasty, otoplasty, nipple reduction, and labioplasty. Analysis of surgical site distribution revealed that the most frequently affected anatomical region was the back (16.9%), followed by the upper extremities (12.1%) and cheeks (10.1%).

**Figure 2:**
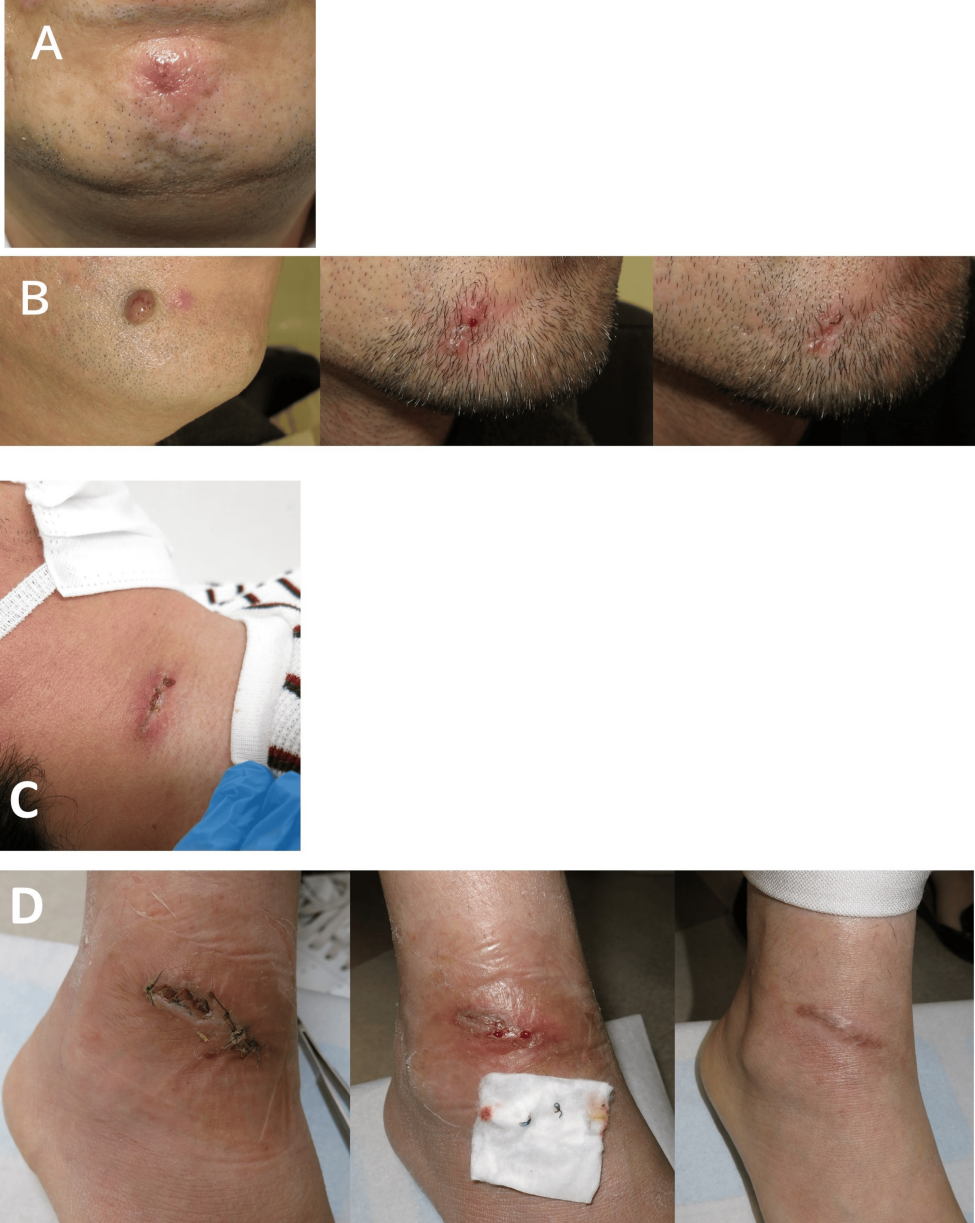
Illustrative cases of postoperative wound infection A (Patient 1): Cellulitis of the male chin following epidermoid cyst excision. B (Patient 2): Nevus of the male chin (left). Cellulitis occurred following excision (center) with clinical resolution demonstrated two-months post-treatment (right). C (Patient 5): Cellulitis and abscess following axillary osmidrosis surgery. D (Patient 6): Wound dehiscence following dermatofibroma excision of the lower leg (left). Subsequent suture abscess (center) with resolution shown one-month post-treatment (right).

During the study period, postoperative wound infections were documented in seven of the 8,742 patients (0.08%), comprising two female and five male patients. Representative case photographs are shown in Figure 2. No statistically significant difference in postoperative infection rates was observed between male and female patients (p = 0.26; OR = 0.33; 95% CI: 0.03-2.02) (Table I). Postoperative wound infections following simple primary closure were identified in five patients, whereas complex surgical procedures were associated with infections in two patients. The incidence of SSI following complex procedures (1.42%) was significantly higher than that associated with simple primary closure (0.06%), with p< 0.01 (OR = 24.7; 95% CI: 2.33-151.7).

Table 2 provides detailed characteristics of the patients who developed SSI. Only one patient (no. 4) had a history of diabetes mellitus; the others had no notable comorbidities. Subcuticular suturing was performed in six patients, with the exception of patient no. 5. Wound cultures revealed the presence of methicillin-sensitive *Staphylococcus aureus* in two patients (nos. 5 and 7). No pathogens were detected in the remaining five patients. All patients were successfully managed with empiric oral antibiotics (oral amoxicillin-clavulanic acid or cefditoren pivoxil) administered for three to seven days with or without early suture removal, resulting in complete wound healing within 10 days.

**Table 2:** Detailed list of patients with surgical site infections Onset, postoperative day; MSSA, methicillin-sensitive *Staphylococcus aureus*; STSG, split-thickness skin graft

Patient no.	Age, sex	Comorbidity	Affected site	Pathology	Procedure	Onset	Culture
1	61, male	No	Chin	Epidermoid cyst	Primary closure	3	-
2	37, male	No	Chin	Nevus	Primary closure	7	-
3	25, female	No	Nape	Epidermoid cyst	Primary closure	4	-
4	53, male	Diabetes	Neck	Epidermoid cyst	Primary closure	6	-
5	32, male	No	Axilla	Osmidrosis	Skin flap	15	MSSA
6	47, female	No	Lower leg	Dermatofibroma	Primary closure	3	-
7	29, male	No	Lower leg	Tattoo	STSG+primary closure	5	MSSA

## Discussion

This retrospective study, encompassing 8,530 procedures, represents one of the largest reports detailing the incidence of SSI in office-based skin surgery. Our overall incidence of SSI (0.08%) was remarkably low compared to recently reported data [4,6,17,18].

Patient-related risk factors

As discussed in the Introduction, both patient- and surgery-related risk factors for SSI in dermatologic and plastic surgery have been extensively evaluated in the literature [5]. A recent meta-analysis by Schlager et al. demonstrated that male sex and immunocompromised patients are significantly associated with higher SSI rates, with diabetic patients also exhibiting a trend toward increased infection risk [4]. No significant associations were identified between SSI risk and smoking, age at surgery, or excision of malignant tumors. Conversely, a large-scale retrospective study by Matos et al. [18], involving 9,031 patients who were not included in the aforementioned meta-analysis [4], reported no significant association between age, sex, smoking, immunosuppression, diabetes, or anticoagulation status, and SSI incidence after office-based skin surgery. In our study, none of the patients who underwent malignant skin tumor excision developed SSI. One patient (no. 4) had diabetes mellitus, whereas the others had no relevant comorbidities. Furthermore, none of the patients who developed SSI were immunocompromised or old. Despite not recommending interruption of antithrombotic therapy, no associated SSIs were observed. These findings highlight the ongoing debate regarding patient-related risk factors for SSI during skin surgery [5].

Five of the seven patients with SSI were male; however, this result was not statistically significant, likely due to the limited number of postoperative infections. Schlager et al. suggested that the health behaviors and compliance of male patients may contribute to an increased risk of postoperative wound infection [4]. However, we propose that the higher risk in male patients may be related to the increased size and secretory activity of sebaceous glands. Giacomoni et al. reported that sebum production in men is approximately twice that in women, and that men possess a broader hair-bearing area and larger pores [19]. Guarch-Pérez et al. recently suggested that skin appendages serve as reservoirs for bacterial colonization, thereby increasing the risk of SSIs [20]. Notably, six of the seven SSIs in our study occurred in hair-bearing regions (male chins, nape, axilla, and lower legs). Of these, five wounds (nos. 1, 2, 3, 6, and 7) were approximated using subcuticular sutures. Ogawa noted that subcuticular sutures in hair-bearing regions could obstruct hair follicles and sebaceous glands at the wound margin, resulting in wound infection [21].

Surgery-related risk factors

Previous studies have frequently evaluated SSI in skin surgery without distinguishing between outpatient and inpatient settings [1,2,5,11]. However, the complexity of surgical procedures in inpatient settings, often involving larger lesions, extended operative times, and general anesthesia, contrasts sharply with the relatively simple procedures typically performed in outpatient settings under local anesthesia or sedation. Postoperative wounds in inpatients are cared for by nurses, residents, or surgeons themselves, whereas outpatient wound care is usually managed by patients themselves or their family members, raising the possibility of suboptimal care, particularly in elderly patients or those undergoing complex procedures. Therefore, providing written postoperative care instructions is crucial, as verbal explanations alone may be insufficient. Nguyen et al. highlighted the importance of instructional handouts in outpatient dermatological surgeries [22]. Although patient adherence to these instructions was not assessed in this study, ensuring proper postoperative care may have contributed to the low SSI rates observed.

The complexity of surgical procedures has been associated with increased SSI rates [1,2,7,8]. An Italian multicenter survey reported SSI incidences of 1.2% for skin tumor excisions, 12.4% for skin grafts, 6.4% for flap surgeries, and 3.1% for breast reconstruction [7]. In the present study, complex procedures, such as skin flaps and grafts, demonstrated a higher SSI risk than simple closures, supporting previous findings. The increased risk of SSI in complex procedures may be attributed to larger defects, longer operative times, and more extensive tissue handling [8].

The use of subcuticular sutures is commonly employed to optimize aesthetic outcomes and prevent dead space [23]. A limited number of studies have demonstrated that subcuticular sutures significantly reduce groin SSI in vascular surgery [24] and abdominal SSI in laparotomy [25] by minimizing subcutaneous hematoma formation. In dermatologic surgery, Rogues et al. reported that patients experiencing hemorrhagic complications exhibited SSI rates ten times higher than those without hemorrhages [17]. Schlager et al. observed that more than 30% of postoperative secondary hemorrhage cases resulted in wound infections following dermatological surgery [2]. These findings indicate that postoperative hemorrhagic complications are strongly correlated with elevated SSI rates. Although the exact incidence is unknown, postoperative hemorrhagic events in our cohort have been exceedingly rare, and none of the SSIs documented in this series appear to have resulted from bleeding. In patient no. 5, an SSI developed two weeks after flap surgery performed without subcuticular sutures. This delayed infection was most likely attributable to wound dehiscence secondary to flap ischemia, although the possible contribution of an underlying hematoma cannot be excluded. Effective techniques, including meticulous tissue handling, precise wound closure, and compression dressings (e.g., elastic bandaging and tie-over dressings), are recommended to mitigate the SSI risk [5,26].

According to the CDC 1999 guidelines, proper surgical techniques are integral to reducing SSI risk [27]. Accordingly, surgical skill and dexterity may influence postoperative wound infection rates. Kulichová et al. reported that wound infections occurred 5.6 times more frequently in surgeries performed by less-experienced surgeons than in those performed by highly experienced surgeons [9]. Recent studies indicate that SSI rates in outpatient skin surgeries performed by dermatologists range from 1.3% to 5.5%, whereas SSI rates reported for general surgeons or general practitioners are considerably higher (7.3%-8.7%) [2]. Conversely, the incidence of SSI in outpatient plastic and aesthetic surgery, including the present study’s findings, is less than 1%, despite the increased complexity of these procedures [6,28]. Notably, a large-scale multicenter study by Nazarian Mobin et al. reported an SSI incidence of 0.06% (3,063 infections in 5,525,255 procedures) for office-based plastic surgeries performed between 2001 and 2012 in the United States [28]. More recently, Wang & Shinder [29] documented an SSI incidence of 0.06% (two cases of infections in 3,129 procedures) in office-based oculoplastic surgeries, and LaGuardia et al. [6] reported a rate of 0.13% (one case of infection in 778 procedures) for office-based pediatric plastic surgery.

Based on these findings, we propose the following recommendations. Surgeons should handle tissues gently and approximate wound edges precisely using fine instruments, preferably under surgical loupe magnification. To minimize hemorrhagic complications, the use of subcuticular sutures and compression dressings is recommended. In hair-bearing regions, subcuticular sutures should be used with caution to reduce the risk of adverse outcomes. Additionally, surgeons should provide comprehensive postoperative care instructions, including clear guidance on recognizing early signs of infection to facilitate timely intervention.

Strengths and limitations

The major strengths of our study include its large sample size, broad age distribution, and the inclusion of various anatomic sites ranging from the scalp to the toes. However, this single-center retrospective study has some limitations. Patients who developed SSI but did not return for postoperative care may have been missed, potentially underestimating the rate of SSIs. Additionally, the patient population was biased toward benign cystic lesions, with fewer complex cases or malignant tumors. Furthermore, although we suggest subcuticular sutures in hair-bearing skin as a potential high-risk factor, this could not be statistically quantified due to inter-individual variations in hair-bearing regions.

## Conclusions

This large retrospective study of 8,742 patients undergoing Class I and II office-based skin surgery demonstrated an extremely low SSI incidence of 0.08%, which was considerably lower than previously reported rates. While most previous studies have emphasized patient-related risk factors and antibiotic prophylaxis, this study emphasized the importance of meticulous surgical techniques, particularly concerning subcuticular sutures and postoperative wound care instructions.

Future studies should focus on prospective, multicenter investigations involving a diverse range of procedures and multiple surgeons to establish evidence-based guidelines for office-based skin surgeries.
